# 

**Published:** 1893-02-25

**Authors:** 


**Medical History from the Earliest Times.** By E. T. WITHINGTON, M.A., M.B.Oxon. XLIV.—Unprofessional Medicine in the Middle Ages . . . . . . . . . . . 341

**Contemporary Theory and Research.**—Progress in Bacteriology—Prehistoric Trepanning . . . . . . . 342

**Diet in Disease.** By Mrs. ERNEST HART. XIII.—Diabetes (*continued*) . . . . . . . . . . . . 343

**Everybody's Page** . . . . . . . . . . . 344

**Annotations.**—Dr. Tibbits' Libel Action—A Blackburn "Hospital Scandal"—Doctors and the Drink Bill . . . 345

**Notes and News** . . . . . . . . . . . 346

**Editor's Letter Box.**—The Mildmay Mission Hospital—The Institute of Preventive Medicine . . . . . . . . 353

**The Hospital Clinic—**

ST. MARY'S HOSPITAL.—The Treatment of Gastric Ulcer . . 347

PADDINGTON GREEN CHILDREN'S HOSPITAL.—The Treatment of Rickets . . . . . . . . . . . . 347

NEWCASTLE-ON-TYNE THROAT AND EAR HOSPITAL.—Treatment of Otorrhœa (Discharge from the Ear) . . . . . 348

NOTES ON NEW DRUGS.—Hypnal or Monochloral-Antipyrin . 349

PRACTITIONERS' BOOKSHELF.—"The Year Book of Treatment" . 350

**The Institutional Workshop—**

WITHIN THE HOSPITALS.—The Cumberland Infirmary, Carlisle . 351

HOSPITAL MEETINGS.—Royal Sea-Bathing Infirmary at Margate 351

THE STORY OF THE INSANE FROM YEAR TO YEAR . . . 352

**Scraps and Gleanings** . . . . . . . . . 354

**Around the Hospitals—Books Received** . . . . 354

**The Student of Medicine.**

**EXTRA SUPPLEMENT.—THE NURSING MIRROR.**

**News from the Nursing World.**—Royalty at Portsmouth—A Plea for the Dolls—A Promising Association—Brave Nurses—Stagnation at Eastville—Interesting and Instructive—Kindly Encouragement—Modern Methods—American Nurses—Proposals in Paris—Short Items—"The Hospital" Endowed Bed—A Disabled Nurse . . . . . . . clvii

**Management of Consumption.** III.—Hæmoptysis . . clix

**Croupous Pneumonia** (*concluded*) . . . . . . clx

**The International Nursing Congress, Chicago.**—Mrs. Bedford Fenwick's Position . . . . . . . clxi

**Girl Nurses Again** . . . . . . . . . . clx

**Everybody's Opinion.**—A Reasonable Request—Provincial Training—Young Nurses—American Experiences . . clxi

**Royal British Nurses' Association** . . . . . clxii

**Appointments—Minor Appointments** . . . . . clxii

**For Reading to the Sick.**—Example . . . . . clxiii

**An American Training School** . . . . . . clxiii

**Notes and Queries—Hints to Nurses** . . . . clxiii

**The Muses' Looking-Glass.**—Burne Jones at the New Gallery . . . . . . . . . . . . clxiv

